# Colony-YOLO: A Lightweight Micro-Colony Detection Network Based on Improved YOLOv8n

**DOI:** 10.3390/microorganisms13071617

**Published:** 2025-07-09

**Authors:** Meihua Wang, Junhui Luo, Kai Lin, Yuankai Chen, Xinpeng Huang, Jiping Liu, Anbang Wang, Deqin Xiao

**Affiliations:** 1 College of Mathematics and Informatics, South China Agricultural University, Guangzhou 510642, China; wangmeihua@scau.edu.cn (M.W.); luojunhui@stu.scau.edu.cn (J.L.); scau_kk@stu.scau.edu.cn (K.L.); ab9853211@stu.scau.edu.cn (Y.C.); anbang1385@163.com (A.W.); 2 Guangdong Provincial Key Lab of Agro-Animal Genomics and Molecular Breeding, College of Animal Science, South China Agricultural University, Guangzhou 510642, China; hxpkakakad@163.com (X.H.); liujiping@scau.edu.cn (J.L.); 3 Key Laboratory of Smart Agricultural Technology in Tropical South China, Ministry of Agriculture and Rural Affairs, South China Agricultural University, Guangzhou 510642, China

**Keywords:** colony detection, mulberry bacterial blight, YOLOv8, StarNet, attention mechanism, loss function

## Abstract

The detection of colony-forming units (CFUs) is a time-consuming but essential task in mulberry bacterial blight research. To overcome the problem of inaccurate small-target detection and high computational consumption in mulberry bacterial blight colony detection task, a mulberry bacterial blight colony dataset (MBCD) consisting of 310 images and 23,524 colonies is presented. Based on the MBCD, a colony detection model named Colony-YOLO is proposed. Firstly, the lightweight backbone network StarNet is employed, aiming to enhance feature extraction capabilities while reducing computational complexity. Next, C2f-MLCA is designed by embedding MLCA (Mixed Local Channel Attention) into the C2f module of YOLOv8 to integrate local and global feature information, thereby enhancing feature representation capabilities. Furthermore, the Shape-IoU loss function is implemented to prioritize geometric consistency between predicted and ground truth bounding boxes. Experiment results show that the Colony-YOLO achieved an mAP of 96.1% on MBCDs, which is 4.8% higher than the baseline YOLOv8n, with FLOPs and Params reduced by 1.8 G and 0.8 M, respectively. Comprehensive evaluations demonstrate that our method excels in detection accuracy while maintaining lower complexity, making it effective for colony detection in practical applications.

## 1. Introduction

Mulberry is an important crop for the silk industry, and its healthy growth is crucial for the stability of the global silk supply chain [1,2]. In recent years, mulberry bacterial blight has become one of the major diseases affecting its growth and yield [3,4]. The recognition of crop diseases based on crop leaf symptoms is a basic method of crop disease detection [5]. However, when mulberry trees exhibit typical disease symptoms, their pathological progression has usually reached the middle to late stages, at which point therapeutic interventions may become more challenging [6]. Pathogenic bacteria are the primary cause of mulberry bacterial blight, which has prompted researchers to focus on the causal pathogens [7,8]. By accurately identifying pathogenic microorganisms and systematically analyzing their infection pathways and pathogenicity factors, experts can not only build a complete theoretical model of disease occurrence, but also provide a key scientific basis for developing targeted prevention and control strategies [9,10]. Colony detection is an important and tedious task in plant disease research [11]. However, traditional methods for manual colony counting, such as the plate counting method and the turbidimetry method, are time-consuming, have low accuracy, and are complex to operate, making them difficult to apply on a large scale [12,13].

Currently, research on intelligent recognition of colonies mainly focuses on traditional machine learning algorithms and deep learning-based methods. Traditional algorithms, such as watershed segmentation, thresholding, distance transforms and wavelet transforms, are widely used for colony counting [14,15,16]. Brugger, S.D. et al. [17] designed a colony hardware and wrote a segmentation algorithm based on Top-Hat filtering and the Bayes classifier. Geissmann, Q. [18] developed an open-source software called OpenCFU, which tests all possible threshold values and retains only the morphologically valid regions that frequently occur to enhance robustness. Yoon, S. et al. [19] used hyperspectral imaging to develop a colony segmentation algorithm for detecting non-O517 Shiga toxin-producing Escherichia coli (STEC) pathogens on Rainbow agar. Zhang, L. [20] combined unsupervised machine learning, iterative adaptive threshold segmentation, and watershed segmentation based on local minima to achieve accurate and robust colony counting. Khan, A.U.M. et al. [21] introduced AutoCellSeg, a MATLAB-based tool that employs an image segmentation method which utilizes multi-thresholding techniques complemented by a feedback-based watershed algorithm. Chen, W. et al. [22] proposed a one-class support vector machine (SVM) counter using radial basis function (RBF) as a classifier in order to differentiate colonies of different bacterial species. 11. Choudhry, P. et al. [11] developed the “Cell Colony Edge” ImageJ macro and the “Cell Colony Counting” CellProfiler pipeline which can be applied to counting cells and colonies, as well as measuring their area, volume, morphology, and intensity. However, the traditional algorithms mentioned above may exhibit low accuracy when dealing with low-resolution images or high noise levels, and the accuracy of colony classification may be suboptimal.

In recent years, researchers have explored the use of deep learning methods for colony counting and have achieved notable results. Carl, S.H. et al. [23] developed a fully automated pipeline for colony segmentation and classification using U-net and ResNet-34. Clarke, M.L. et al. [24] proposed a low-cost, high-throughput colony counting system consisting of colony-counting software and a consumer-grade digital camera or document scanner. Wang, H. et al. [25]. designed a two-step framework for bacterial colony detection. The first step selects candidate colonies by differential image analysis and refines the results with a detection deep neural network. The second step further classifies the detected colonies into species using a classification DNN model with a similar network architecture. Nagy, S.Á. et al. [26] used ADBC to pre-train the Faster R-CNN and estimate the growth rate of S. aureus using the best weights. Cao, L. et al. [27]. developed an automated colony segmentation and counting system using U^2^-Net and obtained 99.5% F1 value on the validation set. Jumutc, V. et al. [28] improved the U-Net by introducing an additional loss term in the neck layer that focuses on auxiliary signaling and reduces the error in colony segmentation. Ebert, N. et al. [29] introduced AttnPAFPN, a high-resolution detection pipeline for AGAR dataset, which employs a novel Transformer variant, efficient global self-attention mechanism. However, the model has a parameter count of 32.8 M, leading to significantly higher computational costs compared to models like You Look Only Once (YOLO).

Object detection method based on YOLO achieved high precision detection while ensuring fast detection speeds, making it widely applicable in object detection tasks. The YOLO has been widely applied in agriculture [30,31] and animal husbandry [32,33,34], and some researchers have used YOLO to detect colonies. Zhang, B. et al. [35] improved the average accuracy from 64.3% to 97.4% by making lightweight improvements to the YOLOv3 network based on a few-shot learning strategy. Ma, L. et al. [36] applied YOLOv4 for the detection of Escherichia coli colonies, enabling accurate identification of E. coli at the microcolony stage after 3 h of cultivation. By integrating with phase-contrast microscopic imaging, YOLOv4 discriminates E. coli from seven other common foodborne bacterial species. Whipp, J. et al. [37] utilized images of Staphylococcus aureus (S. aureus) from the AGAR dataset to compare various YOLOv5 models. The mAP performance measure ranges from 96.1% to 99.1%. The results indicated that more complex models did not lead to significant performance improvements but did considerably increase training time. Notably, this study exclusively utilized a single colony type from the AGAR dataset for model training, which may limit its generalizability to diverse microbial communities. Liu, C [38] designed an improved amyolov5 model based on the YOLOv5 model for the phenomenon of low resolution of small targets of colonies, which leads to serious leakage detection, and developed an automatic colony identification and analysis system based on the micro-service architecture. However, the study acknowledges a limitation: the model demonstrates suboptimal performance in detecting adhered colonies, particularly in scenarios with high-density clustering.

The aforementioned research has proposed numerous methods for tasks such as colony segmentation and detection of a limited number of colony types, achieving some beneficial successes. However, significant challenges remain, such as misdetection of micro-colonies, incorrect distinction of adhered colonies, and high computational costs. Addressing the aforementioned challenges, a colony detection network named Colony-YOLO is proposed. The main contributions of this study are as follows:

(1) A dataset of mulberry blight bacterial colonies named MBCD is proposed, including nine species of bacteria, 310 images, and 23,524 colonies.
(2) The StarNet is deployed as the backbone network for Colony-YOLO. StarNet adopts a model design based on star-shaped operations, significantly enhancing the ability to transform input features into high-dimensional feature spaces while effectively reducing computational complexity.
(3) The C2f-MLCA module is designed to significantly enhance the network’s feature extraction capability by integrating local and global features along with channel and spatial information, thereby improving feature extraction capabilities and detection accuracy.
(4) The Shape-IoU is used as the bounding box regression loss to make the model focus on the shape and scale of the bounding box itself, thereby improving its localization ability.

## 2. Datasets

### 2.1. Mulberry Bacterial Blight Colony Dataset

Colony cultivation and image collection were completed by research team members. The bacteria are isolated from mulberry bacterial blight samples collected in eight provinces of China [4]. The bacteria were cultured from November 2024 to December 2024 at the Guangdong Provincial Key Laboratory of Agro-Animal Genomics and Molecular Breeding, South China Agricultural University. Information on the bacteria species and infections is shown in Table 1.

Colonies were cultured on an ultra-clean bench and ozone incubator. The pure culture of target bacteria was placed in Luria–Bertani (LB) medium (Peptone 10 g·L^−1^, Yeast Extract 5 g·L^−1^, NaCl 5 g·L^−1^, D-Glucose 1 g·L^−1^, pH 7.0) and incubated at 28 °C, 160 rpm with shaking overnight to achieve the fermentation broth of target bacteria. After shaking, samples were subjected to a 10 fold gradient dilution in sterile water, then 100 μL aliquots from 1 × 108 CFU (OD600 = 0.1) gradient were taken and coated on the Luria–Bertani (LB) agar medium (Peptone 10 g·L^−1^, Yeast Extract 5 g·L^−1^, NaCl 5 g·L^−1^, D-Glucose 1 g·L^−1^, Agar 18 g·L^−1^, pH 7.0) Finally, the Petri dish was incubated at 28 °C for 1 to 2 days until colonies grew. After the colonies grew on the medium, the morphological characteristics of the colonies were observed, and the colony images were collected in a photo box.

**Figure 1:**
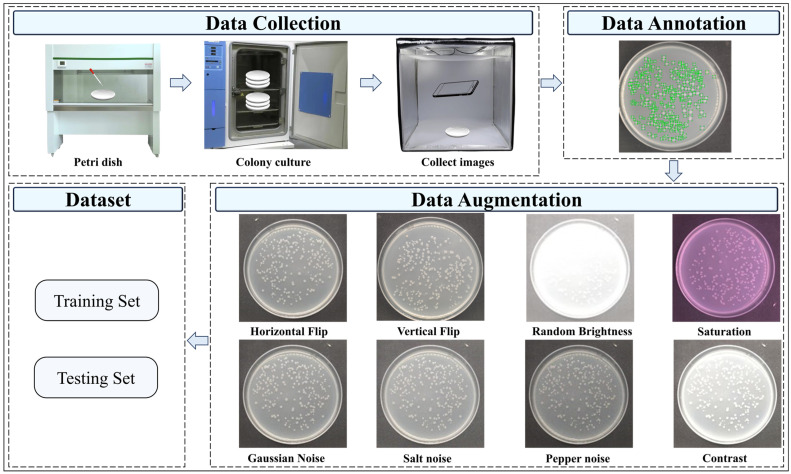
MBCD data collection and augmentation.

**Figure 2:**
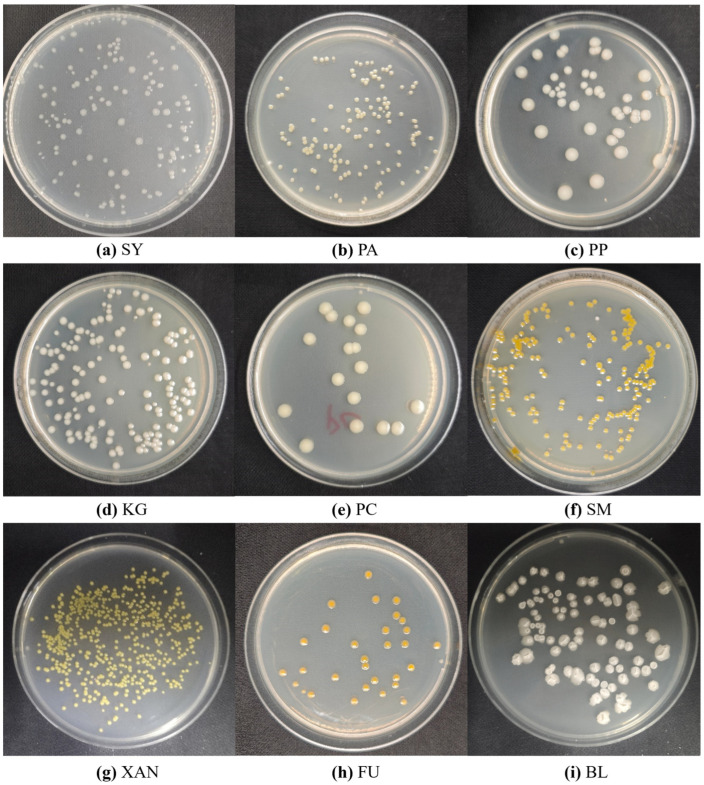
Example raw colony images of nine types of colonies on agar plates of MBCD.

To enhance the quality of the dataset for improved model training, a series of preprocessing steps were applied to the captured images, as shown in Figure 1. Each image was cropped to a resolution of 2250 × 2250 pixels with a 1:1 aspect ratio. Example raw colony images of nine types of colonies on agar plates of MBCD after cropping are displayed in Figure 2. This cropping process aimed to eliminate any extraneous background and retain only the Petri dish, thereby ensuring that the relevant features of the colonies were more prominently displayed.

To further enrich the dataset and introduce greater variability, multiple data augmentation techniques were employed on the original images, as shown in Figure 1:

(1) Performing horizontal or vertical flips to create mirrored versions of images significantly increases the diversity of the dataset, allowing the model to learn features of colonies from different directions, thereby enhancing its ability to recognize them from various angles.
(2) Randomly adjusting brightness, contrast, and saturation allows the model to better adapt to varying lighting conditions and image qualities during training.
(3) Adding noise to simulate different environmental conditions. Gaussian noise, salt noise, and pepper noise can replicate the noise interference encountered when capturing images in real environments, thus improving resilience to noise, enhancing model performance in complex scenarios.

Labeling of colonies was performed using LabelImg, and the information containing the category and location of colonies was stored in txt file. The dataset after processing consists of nine distinct types of colonies, encompassing a total of 310 images and 23,524 individual colonies, which is strategically divided into a training set containing 248 images and a validation set with 62 images, following an 8:2 ratio. The colony distribution in images is highly dense, with a single image typically containing dozens to hundreds of instances of colonies. Such rich colony instances in images mean that only a small number of images are required to meet the training requirements of the model.

### 2.2. Annotated Dataset for Deep-Learning-Based Bacterial Colony Detection

**Figure 3:**
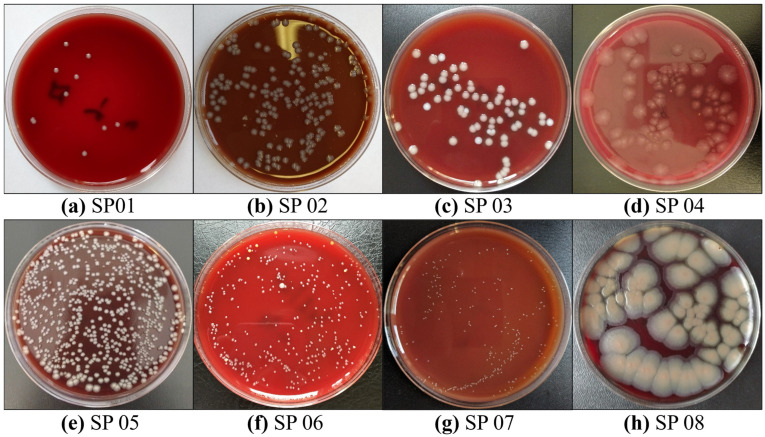
Example raw colony images of eight bacterial species on agar plates of ADBC.

The Annotated Dataset for Deep Learning-Based Bacterial Colony Detection (ADBC) [47] was proposed by Makrai, L. et al. This dataset is specifically designed to aid in the development and evaluation of deep learning models for detecting bacterial colonies. It consists of 369 images, totaling 24 distinct types of colonies. A meticulous annotation process was carried out, resulting in the manual labeling of 56,865 individual colonies. All annotations were created using the maximum horizontal bounding box approach, ensuring the precise localization of each colony within the images. The images were provided in JPG format, and the annotation information was stored in TXT files. Similarly, the dataset was divided into a training set of 292 images and a validation set of 75 images at 8:2. The data of eight types of colonies are shown in Figure 3.

## 3. Methods

### 3.1. YOLOv8 Network

The YOLOv8 object detection network was introduced by the Ultralytics in 2023, offering five versions: n, s, m, l, and x. The depth and parameters of YOLOv8 increase progressively from n (lightest) to x (most complex), with a corresponding improvement in detection accuracy. Considering the real-time requirements and computational resource constraints in practical application scenarios, this study selects YOLOv8n, the version with the shallowest network depth, the fewest parameters, and the fastest inference speed, as the baseline model for algorithm improvement.

The YOLOv8n network architecture consists of three components: backbone, neck, and head network [48]. The backbone network is constructed based on the Conv module, which comprises Conv2d, Batch Normalization (BN) and SiLU activation function for preliminary feature extraction. Concurrently, the Cross-Stage Partial Structure with 2 Convolutional Operations (C2f) module is integrated to optimize feature reuse and gradient propagation via a hierarchical process feature transformation, branch processing and feature fusion. Additionally, the improved spatial pyramid pooling fast module (SPPF) is integrated to efficiently capture multi-scale contextual information using multi-scale max-pooling operations (e.g., 5 × 5, 9 × 9, 13 × 13), thereby expanding the model’s receptive field. The input size of YOLOv8 is 640 × 640, and the original image needs to be resized to the standard size before being input into the network. Considering that direct stretching may cause target proportion imbalance (distortion), YOLOv8 follows the preprocessing strategy of YOLOv5: first perform proportional scaling on the image (adjusting the width or height to 640 pixels), and then use background padding to unify the image size to 640 × 640. The neck network adopts a bidirectional feature pyramid architecture including FPN [49] and PAN [50], which transmits high-level semantic information top-down through FPN and fuses low-level detailed features bottom-up via PAN, supplemented by channel-wise concat operations to concatenate feature maps from different stages, achieving collaborative optimization of low-level high-resolution features and high-level strong semantic features. The head network employs decoupled head design to separate classification and bounding box regression tasks, eliminating feature interference. It also utilizes an anchor-free mechanism to directly predict target center point offsets and width–height parameters, abandoning the limitations of traditional preset anchor boxes, significantly reducing parameter quantity, and enhancing generalization capabilities for dense small targets.

### 3.2. Overall Structure of Colony-YOLO

**Figure 4:**
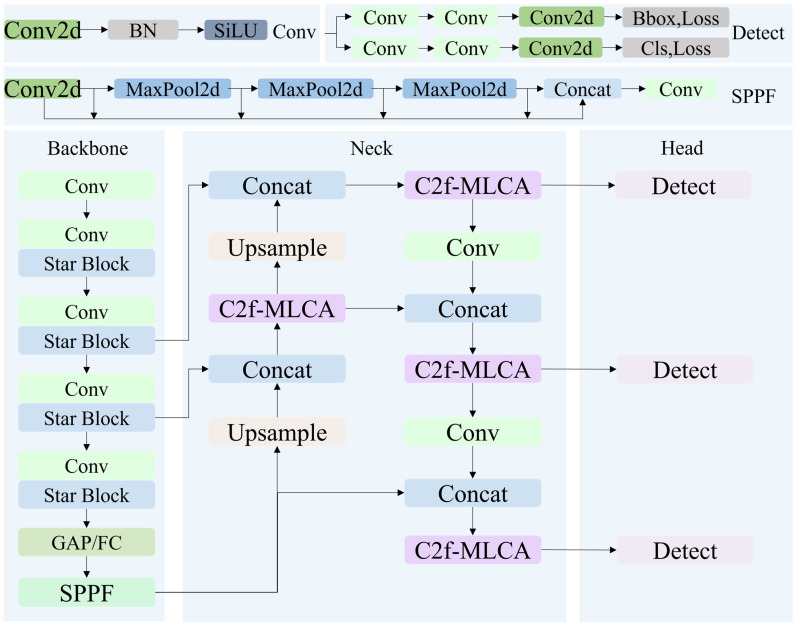
Structure diagram of Colony-YOLO. The Conv block is composed of convolution2d, BN, and the SiLU activation function. Spatial Pyramid Pooling Fusion (SPPF) is composed of multi-scale pooling layers, a feature fusion module, and convolution layers. GAP/FC is global average pooling or fully connected layer.

To address the challenges of detecting micro-colonies and densely distributed colonies in complex scenarios, Colony-YOLO is optimized and improved in the following aspects: (1) the StarNet is employed as the backbone network of Colony-YOLO; (2) the C2f-MLCA module is designed and applied to the neck network of the model; and (3) the Shape-IoU loss function is used. The overall structure is illustrated in Figure 4, providing a visual representation of how these components work together to enhance colony detection performance.

#### 3.2.1. Lightweight Backbone Network StarNet

YOLOv8 adopts CSPNet as its backbone network architecture. While this choice enhances the capability to represent image features, the increased depth of the network structure prolongs both model training and inference times. Notably, in micro-colony detection, spatial resolution sensitivity is crucial for capturing tiny morphological details. The deep hierarchy of CSPNet may introduce feature downsampling, risking the loss of fine-grained spatial information, whereas StarNet’s design circumvents this issue.

To improve colony detection accuracy while reducing model computational costs to meet mobile application deployment needs, this study employs StarNet [51] as a backbone network. StarNet utilizes star operations and Depthwise Separable Convolutions (DWConv) to retain high spatial resolution through shallow yet dense feature extraction. Specifically, star operations enhance local-global feature integration, enabling the model to better distinguish micro-colonies from background noise, while DWConv reduces channel-wise computation without compromising spatial detail sensitivity. This design ensures the model maintains high sensitivity to micro-colony shape and scale, significantly enhancing its nonlinear mapping capability to high-dimensional feature spaces while reducing computational complexity.

The backbone of Figure 4 depicts the structure of StarNet. It starts with the first convolutional layer for feature extraction of the input image. Following this is the holistic hierarchical network framework, which consists of four feature extraction stages called “star stage”. Each “star stage” is composed of a convolutional layer and multiple star blocks. The convolutional layer downsamples the input features while simultaneously increasing the number of channels.

**Figure 5:**
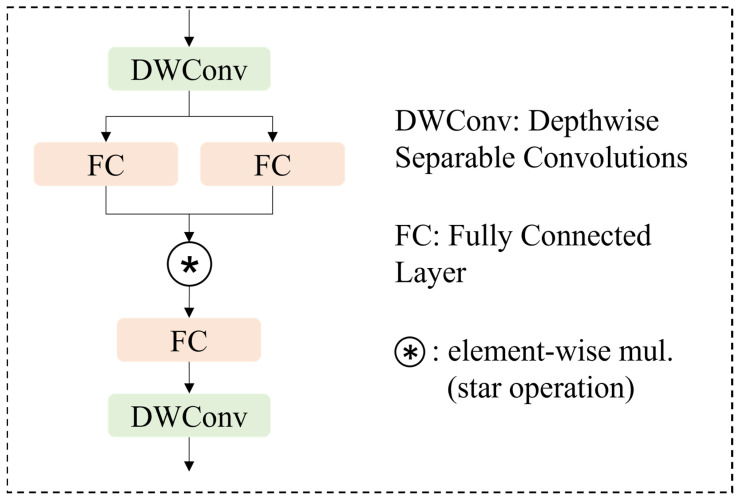
Structure of star block.

The structure of the star block is shown in Figure 5. The star block comprises a DWConv layer, a fully connected layer, and the star operation. The star operation integrates features from two branches through element-wise multiplication. Finally, global average pooling or a fully connected layer is applied to consolidate the features, setting the stage for the classification task.

In a single-layer network, the star operation is typically denoted as $\left(W_{1}^{T}X+B_{1}\right)*\left(W_{2}^{T}X+B_{2}\right)$ Incorporating the weight matrix W and bias B into a unified entity, denoting it as $w=\left[\begin{array}{c} W\\ B \end{array}\right]$. Correspondingly, the input vector X is expanded into a matrix $x=\left[\begin{array}{c} X\\ 1 \end{array}\right]$. In this manner, the star operation implemented by StarNet can be represented as:

(1) $$W_{1}^{T}x*W_{2}^{T}x=\frac{\alpha_{\left(1,1\right)}x^{1}x^{1}+\ldots +\alpha_{\left(d+1,d+1\right)}x^{d+1}x^{d+1}}{(d+2)(d+1)/2}$$

(2) $$\alpha_{\left(i,j\right)}=\left\{\begin{array}{ll} w_{1}^{i}w_{2}^{j}, & i=j\\ w_{1}^{i}w_{2}^{j}+w_{1}^{j}w_{2}^{i}, & i\neq j \end{array}\right.$$

where i and j index the channels and α denotes the coefficient for each item. d represents the number of input channels.

According to Equation (1), apart from the special terms, each remaining term exhibits a nonlinear relationship with x, indicating that these terms represent independent, implicit dimensions. The star operation is expanded into ${\left(\frac{d}{\sqrt{2}}\right)}^{2}\left(d\gg 2\right)$ independent components, significantly amplifying the feature dimensions without incurring additional computational overhead within a single layer. This principle allows for high-dimensional feature mapping in low-dimensional space and moving away from the traditional method of increasing expressive power by adding more channels. As a result, it not only enhances the model’s ability to extract and represent complex features effectively, but also significantly reduces computational costs, leading to a more lightweight model.

#### 3.2.2. C2f-MLCA

In colony detection tasks, colony targets typically exhibit characteristics of small size, diverse shapes, and dense distribution. Additionally, the complex textures of the agar medium background and interference from impurities further escalate the detection challenges. Although the C2f module achieves feature reuse and efficient fusion through inter-stage partial connections, there is still scope for enhancing its collaborative perception of local details and global context. In this study, the C2f-MLCA module is designed to enhance the collaborative perception of local details and global context of colony feature while retaining the feature reuse advantages of the C2f structure.

**Figure 6:**
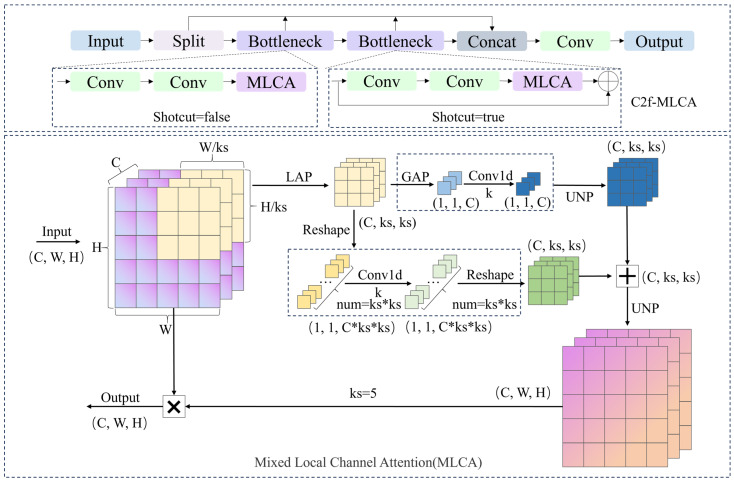
Structure of MLCA and C2f-MLCA. UNP denotes unpooling.

The module structures of MLCA [52] and C2f-MLCA are illustrated in Figure 6. MLCA significantly enhances the network’s ability to recognize and capture critical features by integrating local and global features, as well as channel and spatial information. In the initial stage, MLCA collects local spatial details through local average pooling (LAP), resulting in a feature map of size (1×C×ks×ks) where C represents the number of channels and ks indicates the size of the pooling window. Following this, the feature map is divided into two streams: one stream extracts global context through global average pooling (GAP), while the other is used to preserve local spatial information. The GAP features are processed through reshaping, conv1d, and unpooling. The conv1d compresses feature channels while preserving spatial dimensions. In MLCA, the size k of the Conv1d convolution kernel is directly proportional to the channel dimension C, aiming to capture local cross-channel interaction information by only considering local features between each channel and its K adjacent channels. The selection of k is determined by Equation (3).

(3) $$k=\Phi \left(C\right)={\left|\frac{{log}_{2}\left(C\right)}{\gamma }+\frac{b}{\gamma }\right|}_{odd},\;\gamma =2,b=2\;,$$

where γ and b are hyperparameters with default values of 2; odd indicates that k takes only odd values. If C is even, it is incremented by 1 before the calculation.

The LAP features, after reshaping, conv1d processing, and reshaping once again, are combined with the processed GAP features, enabling the feature map to integrate global context information effectively. Finally, the feature map, which fuses both local and global attention, is restored to the original spatial dimensions through unpooling and element-wise multiplied with the original feature map. This process not only preserves the integrity of the features but also enhances their representational capacity. Therefore, the design of the MLCA module significantly improves the network’s detection accuracy by combining channel attention and spatial attention at both local and global levels, while maintaining computational efficiency.

Building on this design, the MLCA is integrated into the C2f module, which not only strengthens the capture of crucial features such as colony target edges and textures, but also improves the localization accuracy of small and densely distributed targets through global context information. The C2f-MLCA module employs lightweight attention design, making the increased computational cost negligible, thereby optimizing detection performance while maintaining inference efficiency.

#### 3.2.3. Shape-IoU Loss Function

**Figure 7:**
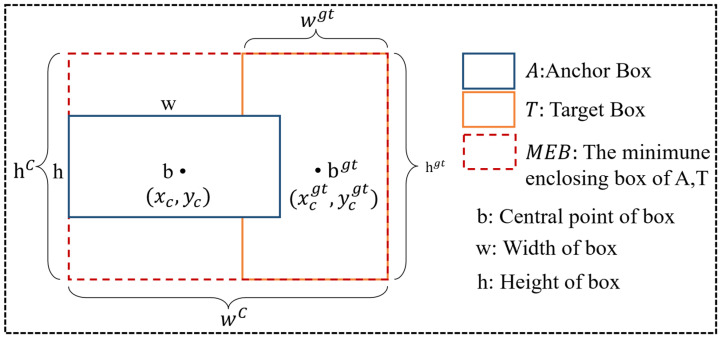
Anchor bounding box and target bounding box location information.

The loss function serves as a critical metric for measuring the difference between predicted results and ground truth labels, with its value decreasing as the predictions approach the true labels. Traditional losses like CIoU, DIoU, or GIoU treat all edge errors equally and ignore intrinsic properties such as the shape and scale of the bounding boxes, which can impact regression performance. To address the issue of insufficient localization accuracy for micro-colonies in colony images, the loss function is replaced with Shape-IoU [53], aiming to resolve the insensitivity of the CIoU loss function to variations in the scale and shape of bounding boxes. Shape-IoU calculates loss by focusing on the shape and scale of bounding boxes, thereby improving the accuracy of bounding box regression. The anchor bounding box and target bounding-box location information positional information in Figure 7. The functions of Shape-IoU are as follows:

(4) $$L_{IoU}=1-\frac{\left|A\cap T\right|}{\left|A\cup T\right|},$$

(5) $${weight}_{w}=\frac{2\times {\left(w^{gt}\right)}^{scale}}{{\left(w^{gt}\right)}^{scale}+{\left(h^{gt}\right)}^{scale}},$$

(6) $${weight}_{h}=\frac{2\times {\left(h^{gt}\right)}^{scale}}{{\left(w^{gt}\right)}^{scale}+{\left(h^{gt}\right)}^{scale}},$$

(7) $${distance}^{shape}={weight}_{h}\times \frac{{\left(x_{c}-x_{c}^{gt}\right)}^{2}}{c^{2}}+{weight}_{w}\times \frac{{\left(y_{c}-y_{c}^{gt}\right)}^{2}}{c^{2}},$$

(8) $$\Omega^{shape}=\sum_{t=w,h}{\left(1-e^{-\omega_{t}}\right)}^{\theta },\theta =4,$$

(9) $$\left\{\begin{array}{c} \omega_{w}={weight}_{h}\times \frac{\left|w-w^{gt}\right|}{\max\left(w,w^{gt}\right)}\\ \omega_{h}={weight}_{w}\times \frac{\left|h-h^{gt}\right|}{\max\left(h,h^{gt}\right)} \end{array}\right.,$$

(10) $$L_{Shape-IoU}=L_{IoU}+{distance}^{shape}+0.5\times \Omega^{shape},$$

where ${weight}_{h}$ and ${weight}_{w}$ represent weight coefficients in the vertical and horizontal directions, whose values depend on the configuration of the ground truth box. Scale denotes the scaling factor, which is related to the scale of colonies in the dataset. ${distance}^{shape}$ and $\Omega^{shape}$ correspond to the shape distance loss and shape dissimilarity penalty term in the loss function, respectively.

In colony detection tasks, Shape-IoU can enhance model detection performance, especially demonstrating unique advantages in identifying small and complex-shaped colonies. Colonies are typically small in size and diverse in shape. Traditional IoU loss functions tend to overlook shape and scale information, leading to issues such as missed detections or inaccurate bounding box fitting when dealing with small or overlapping colonies. Shape-IoU, on the other hand, by introducing distance and shape losses, can more accurately measure the matching degree between the predicted and ground truth boxes. It guides the model to focus more on the geometric features of colonies, thereby optimizing the bounding box regression process. In cases of colony overlap or adhesion, Shape-IoU can reduce false positives and false negatives through the shape dissimilarity penalty term.

## 4. Experiments and Results

### 4.1. Experiment Environment and Configuration

The computational infrastructure for this study comprised an NVIDIA GeForce RTX 3090 GPU with 24 GB VRAM, coupled with an Intel Core i9-9900k processor (3.6 GHz base clock, 16-core architecture) and 32 GB DDR4 RAM. Python package management was carried out through conda 4.9.2.

The deep learning framework was implemented in Python 3.8 using PyTorch 1.12.0, with CUDA 12.6 and cuDNN 8.2.0 acceleration libraries for GPU-accelerated computations. The critical hyperparameter settings are detailed in Table 2.

### 4.2. Evaluation Metrics

The following metrics are used to evaluate the model: precision (P), recall (R), mean average precision (mAP), number of parameters (Params), gigaflops per second (FLOPs), and model size. Precision refers to the proportion of true positives among the detected bounding boxes by the model. Recall refers to the proportion of true positives detected by the model out of all true positives. mAP is the average of average precision AP across all categories, providing a comprehensive reflection of the model’s overall performance across all categories.

(11) $$P=\frac{TP}{TP+FP}$$

(12) $$R=\frac{TP}{TP+FN}$$

(13) $$AP=\int_{0}^{1}P\cdot \left(R\right)dR$$

(14) $$mAP=\frac{\sum_{i=1}^{N}AP_{i}}{C}$$

(15) $$Params=C_{in}*C_{out}*K*K$$

where true positive (TP), false positive (FP), and false negative (FN) represent the number of correctly predicted, falsely predicted, and missed targets. C denotes the number of classes. $C_{in}$ and $C_{out}$ indicate the number of input and output feature channels, respectively. K represents the kernel size.

### 4.3. Ablation Experiment of Colony–YOLO

This study enhances YOLOv8n by incorporating the lightweight backbone network StarNet, C2f-MLCA, and Shape-IoU loss function. To systematically validate the performance improvements of the proposed model, a comprehensive step-by-step comparative analysis was conducted in MBCD between the enhanced model and the baseline YOLOv8n. All ablation experiments were conducted under the same dataset and training parameters, with the detailed comparative results presented in Table 3.

As shown in Table 3, the original YOLOv8n model achieves a mAP of 91.3% on the MBCD for colony detection. Its limitations primarily manifest in challenges such as the susceptibility of micro-colony features to background interference in complex cultivation environments and insufficient localization accuracy for densely distributed targets. Meanwhile, for practical application scenarios, it is also necessary to make lightweight improvements to the model to enhance the speed of colony image detection and reduce the model size. To address these issues, a lightweight backbone network, StarNet, was first introduced. Its compact and simple network structure, along with star operations, increased the mAP to 91.4%, a 0.1% improvement over YOLOv8n. Concurrently, FLOPs decreased by 1.6 G, parameters decreased by 0.79 M, and the weight’s file size decreased by 1.6 MB. Furthermore, MLCA was integrated into the Backbone block of C2f to form the C2f-MLCA module, which was applied to the neck layer of YOLOv8. This resulted in a 1.4% improvement in mAP, reaching 92.7%, without increasing the model’s computational load or weights size. Finally, the Shape-IoU loss function was adopted to replace the original loss function of YOLOv8 to resolve the insensitivity of the CIoU loss function to variations in the scale and shape of bounding boxes, driving the mAP to 91.6%, a 0.3% improvement over YOLOv8n.

To further validate the improvements to the model, we conducted ablation experiments by combining various modules as M4-M7. M4 denotes the incorporation of StarNet and C2f-MLCA, resulting in a 3.2% increase in mAP to 94.5%, showing improvements compared to both M1 and M2 while also reducing FLOPs, parameters, and weights. M5 and M6 demonstrate the effect of Shape-IoU on models that only incorporate StarNet and C2f-MLCA. After replacing the original loss function of YOLOv8 with Shape-IoU, the mAP@50 for the models that only introduced StarNet and C2f-MLCA improved to 93.8% and 95.4%, respectively. M7 showcases the final model Colony-YOLO, which achieved a peak mAP of 96.1%, while also minimizing the model’s FLOPs, parameters, and weights file size to 6.5 G, 2.21 M, and 4.7 MB.

Ablation experiments demonstrate significant synergistic effects among the proposed improvements, validating the effectiveness of the technical approach in this study for colony detection tasks.

### 4.4. Comparative Experiments

#### 4.4.1. Analysis of Lightweight Improvements in Feature Extraction Backbone Networks

Considering the high computational demands and numerous parameters of the YOLOv8n model, it occupies significant memory and reduces operational efficiency, thereby limiting its effective deployment on devices. To ensure that the colony detection model meets practical application needs, this study implements lightweight improvements based on YOLOv8 and conducts relevant experiments. Using YOLOv8 as the base model, MobileNet, Shufflenet, FasterNet [54,55,56], and StarNet are employed as backbone networks to comparison of the mAP, FLOPs, Params, and weights of these four models.

The result of quantitative evaluation of different backbone networks in the MBCD is shown in Table 4. The StarNet achieves the best performance on mAP, reaching 91.4%, while also minimizing the model’s FLOPs, parameters, and weights file size to 6.5 G, 2.22 M and 4.7 MB, showing varying degrees of improvement compared to the MobileNet, ShuffleNet, and FasterNet. MobileNet achieves the second-best mAP (excluding the original YOLOv8n). However, it has the largest FLOPs, parameters, and weights, which are 22.5 G, 8.72 M, and 16.7 MB, respectively. This indicates that MobileNet improves detection performance at the cost of increased network complexity. Compared to MobileNet, ShuffleNet and FasterNet exhibit lower detection performance and achieve mAPs of 84.6% and 86.7%, respectively, while they also have lower FLOPs, parameters, and weights.

Overall, StarNet performs the best in colony detection tasks. Moreover, StarNet has a simple lightweight structure. This characteristic allows StarNet to enhance detection accuracy while maintaining high computational efficiency. Therefore, StarNet is a more suitable backbone network for the colony detection task.

#### 4.4.2. Analysis of Loss Function Comparison Results

The limitation of CIoU lies in its equal treatment of all edge errors, disregarding intrinsic properties such as shape and scale, which may adversely affect regression performance. To address the aforementioned limitations, this study undertook a systematic investigation by replacing several alternative loss functions: SIoU, GIoU, EIoU [57,58,59] and Shape-IoU. Comparative experiments were conducted to evaluate the performance of these loss functions, aiming to identify the most effective option for enhancing the detection capabilities of colony.

As shown in Table 5, the Shape-IoU loss function performs the best on mAP, reaching 91.6%, with the highest P and R metrics of 90.5% and 91.1%, respectively, which are improvements of 0.3%, 0.9%, and 0.3% compared to CIoU. In contrast, although SIoU and EIoU achieve mAP metrics of 90.9 and 90.1, which are close to CIoU, their overall performance is slightly inferior to Shape-IoU, where the mAP values are 0.7% and 1.5% lower than Shape-IoU. GIoU achieves the lowest mAP value of 86.7%, which is 4.9% lower than Shape-IoU. The results mentioned above indicates that Shape-IoU can more effectively optimize the model during bounding box regression, especially when dealing with colonies of diverse shapes.

#### 4.4.3. Comparison of Model Performance on ADBCs and MBCDs

In this study, five mainstream target detection models are selected for comparison, including Faster R-CNN, YOLOv5n, YOLOv8n, YOLOv10n, and the improved model Colony-YOLO proposed in this paper. These models were evaluated on ADBC and MBCD, and the comparative experimental results are presented in Table 6.

The experimental results show that the Colony-YOLO model exhibits advantages in all evaluation metrics. As shown in the comparative experimental results on the private dataset MBCD, the FLOPs and Params of Faster R-CNN are 165.9 G and 58.72 M, which exceed those of Colony-YOLO by 159.4 G and 56.91 M. The higher computational requirements of Faster R-CNN limit its deployment on resource-constrained devices. In comparison with YOLOv5n, the results indicate that the FLOPs of Colony-YOLO are slightly higher than those of YOLOv5n, by 1.1 G, but the Params are lower by 3.5 M. Meanwhile, the mAP of Colony-YOLO has improved by 12% compared to YOLOv5n, with increases in both P and R values. This indicates that Colony-YOLO achieves a balance between model detection performance and computational resource consumption. When compared to YOLOv10n, the results show that the mAP of Colony-YOLO has increased by 4.9%, while the FLOPs and Params have decreased by 0.6 G and 5.4 M, respectively, indicating that the overall performance of Colony-YOLO is better than that of YOLOv10n.

Compared to the baseline YOLOv8n, the mAP metrics of Colony-YOLO of the public dataset ADBC and private dataset MBCD reach 91.1% and 96.1%, which are 4.4% and 4.8% higher, respectively. This significant improvement underscores the effectiveness of the enhancements made in Colony-YOLO. In addition to its superior detection performance, Colony-YOLO also achieves a remarkable Params of only 2.21 M, which are 0.8 M and 0.82 M lower than YOLOv8n making it the most efficient model among those evaluated. Furthermore, Colony-YOLO records the FLOPs of 6.5 G, which is 1.7 G lower than YOLOv8n. This combination of high performance and lightweight characteristics indicates that Colony-YOLO not only optimizes model detection capabilities, but also exhibits good lightweight characteristics.

#### 4.4.4. Visualization of Model Detection

To validate the detection performance of Colony-YOLO, a selection of images was randomly chosen for detection and compared with YOLOv8n. The images contain factors that interfere with colony detection, such as densely adherent colonies, micro-colonies, and background interference from Petri dishes.

**Figure 8:**
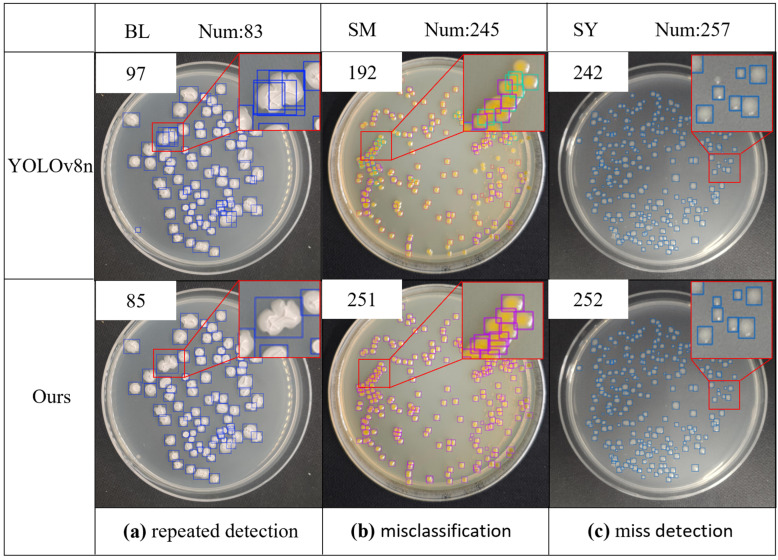
Visualizations of experimental results of private dataset MBCD. Each image is labeled with the predicted results.

Visualizations of experimental results of private dataset MBCD are shown in Figure 8. BL is a type of endophytic fungal colony from mulberry trees that exhibits significant morphological variation. YOLOv8n is affected by colony morphology, resulting in a significant number of duplicate detections for BL, with the actual detection count exceeding the ground true by 14. In contrast, Colony-YOLO performed better, with only two instances of duplicate detections. This indicates that Colony-YOLO can more accurately identify the same type of colony when dealing with morphologically diverse colonies, thereby reducing the occurrence of duplicate detections. The SM type exhibits issues such as dense adherent colonies, indistinct boundaries, and overlapping colonies, all of which may affect the model’s learning effectiveness. Detection results of YOLOv8n included false positives and missed detections, identifying only 192 SM colonies. In contrast, Colony-YOLO had no false positives or missed detections, with only six duplicate detections. This indicates that Colony-YOLO has a significant advantage in detecting adherent colonies. SY consists of colony images captured under low-light conditions. YOLOv8n misses detecting 15 colonies of SY, while Colony-YOLO misses detecting only 5 colonies. The miss detection rate of Colony-YOLO is lower than that of the YOLOv8n model.

**Figure 9:**
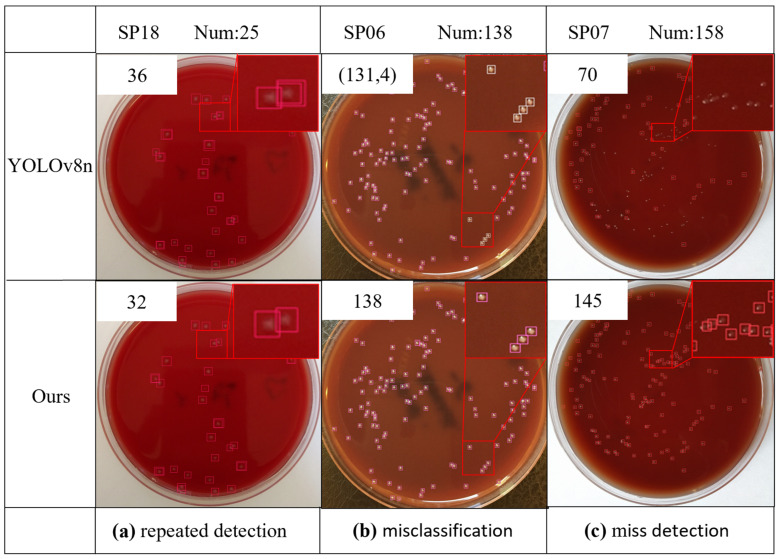
Visualizations of experimental results of public dataset ADBC. Each image is labeled with the predicted results. (131,4) means that there are four colonies that are misidentified as other species of colonies.

The experimental results of the public dataset ADBC are illustrated in Figure 9. The background interference in the ADBC dataset’s Petri dishes is significant, which increases the difficulty of model detection. SP18 is severely affected by the red background. The SP18 color is transparent, and the colonies are small, making them less distinguishable in the red background culture dish, which leads to duplicate detections in the detection results. Detection results of YOLOv8n included duplicate detections of 11 colonies. In contrast, Colony-YOLO performed better, with only seven instances of duplicate detections. In the detection of SP06, YOLOV8n encountered instances of misclassification, while the detection results of Colony-YOLO were consistent with the ground truth. SP07 contains micro-colonies under the red background. YOLOv8n failed to detect 88 colonies, while Colony-YOLO only failed to detect 13 colonies. This indicates that Colony-YOLO has a stronger capability for detecting micro-colonies and reduces the background’s impact on the ADBC dataset, thanks to the improved feature extraction from its C2f-MLCA module.

In summary, Colony-YOLO demonstrated significant advantages in detection performance compared to YOLOv8n under various challenging conditions. The analysis of the experimental results of MBCD demonstrated that Colony-YOLO effectively minimized duplicate detections, particularly in morphologically diverse colonies like BL. Additionally, Colony-YOLO outperformed YOLOv8n in detecting densely adherent colonies (SM), showcasing its robustness against background interference. Results from the public dataset ADBC further indicate that Colony-YOLO effectively addresses the challenges posed by background factors, demonstrating excellent detection performance for both micro-colonies and adherent colonies.

## 5. A Smartphone App for Colony Detection

**Figure 10:**
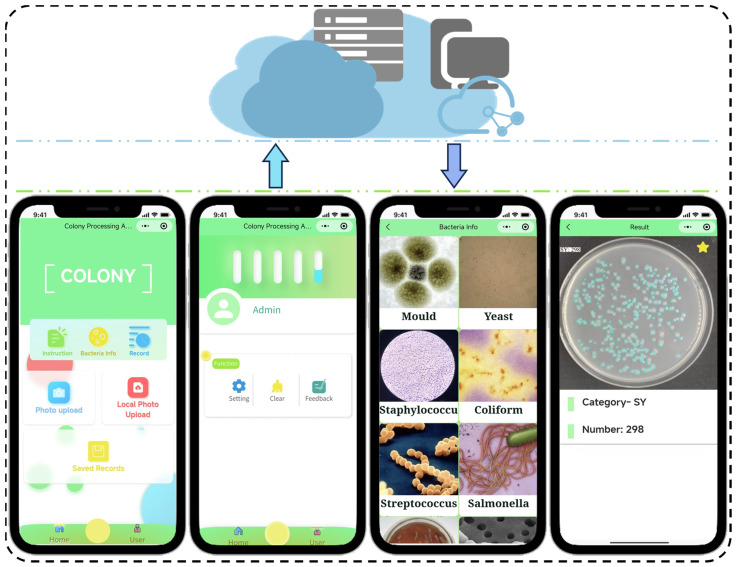
Schematic of the Colony Counting Platform. Images are uploaded via the user’s smartphone network to the cloud, where the platform processes and analyzes the colonies, displays bacterial information, and provides colony type and count results.

To facilitate the practical application of intelligent colony detection technology in mobile scenarios, this study designed and developed a cross-platform intelligent detection system based on the Colony-YOLO model. The proposed system enables real-time image acquisition and processing on mobile devices, automatically detecting and counting colonies, thus providing a convenient and efficient solution for rapid on-site detection. Figure 10 presents the core architecture of the application. Through an interactive interface, users can either capture images in real time or import them from the local gallery. The system supports image processing in popular formats such as JPG and PNG. Once selected, images can be uploaded to the cloud server for further analysis. After selecting an image, it is uploaded to the cloud server for processing. Upon completion, the colony count results and the annotated image are displayed on the results page. Additionally, all count records are automatically stored in the history log, enabling users to access and review past results at any time.

## 6. Discussion

### 6.1. The Advantages of the Proposed Approach

This study addresses challenges in colony detection of the pathogen causing mulberry bacterial blight by proposing the Colony-YOLO model based on improved YOLOv8n, which enhances detection accuracy and reduces the computational consumption. Traditional methods often rely on manual counting or conventional machine learning approaches, which are susceptible to subjective errors, low-resolution images, and interference from complex backgrounds. By introducing a deep learning object detection framework and optimizing it for colony characteristics, Colony-YOLO not only overcomes the inefficiencies and subjectivity of manual detection but also enhances the model’s sensitivity to micro-colonies. Particularly in the detection of the colony of mulberry bacterial blight.

Colony-YOLO enhances the backbone network of YOLOv8n by incorporating StarNet, which improves feature extraction capabilities while reducing computational overhead, making the model more suitable for deployment in mobile apps to align with practical application scenarios. Additionally, the C2f-MLCA module is designed and applied to the neck network of the model, further enhancing the network’s feature extraction capability and its performance in colony detection. Finally, the Shape-IoU loss function is introduced, utilizing its characteristics that focus on the shape and size of the bounding boxes to calculate loss, resulting in more accurate bounding box regression. Experimental results indicate that Colony-YOLO achieves superior detection performance on ADBC and MBCD. This improvement is primarily attributed to optimizations in the model’s ability to detect small targets, feature representation, and localization accuracy. Colony-YOLO exhibits significant application potential in microbiological analysis, assisting researchers in quickly analyzing microbial culture results and enhancing experimental efficiency in laboratory settings.

### 6.2. Analysis of Limitations and Future Work

This study has improved detection performance through the aforementioned enhancements to the model. However, the model still experiences false positives and missed detections. On one hand, extremely small colonies like SY and SP07 exceed the model’s detection limits, making them visually difficult to identify. As shown in Figure 9 and Figure 10, Colony-YOLO recorded 7 missed detections for SY and 13 missed detections for SP07. On the other hand, colonies may not be accurately identified by the model due to overlapping or adhesion, such as BL and SM. Colony-YOLO recorded two duplicate detections for BL and five duplicate detections for SM. Additionally, the background color of the culture dish can also affect model training and detection results. Colony-YOLO encountered duplicate detections of 11 colonies in the detection results for SP18. Future research can adopt the following strategies: first, diversify the training data by incorporating samples of micro-colonies, adherent colonies, and a broader range of bacterial colony types. This initiative aims to enhance the recognition of micro-colonies, improve the discrimination of adherent colonies, and expand the model’s capability to identify unknown colony morphologies. Second, draw on techniques like semantic segmentation to better handle overlapping or adhered colonies, thereby improving the model’s accuracy in complex scenarios. Additionally, reflections on the Petri dishes and factors related to the capturing equipment can impact model training during data collection. In future research, it is essential to utilize higher-performance imaging devices and optimize the data collection process to obtain higher-quality datasets. Finally, it should be noted that the reported performance metrics (precision, recall, mAP) are based on a single validation, which may lead to variance in evaluation results. Although this approach is consistent with the conventions of YOLO-based object detection studies [31,32,33,34,35,36,37,38,39], future work could introduce repeated splits or five-fold cross-validation to provide confidence intervals, thereby enhancing the statistical robustness of performance claims.

## 7. Conclusions

To address the challenges in detecting colonies of mulberry bacterial blight, such as tiny colony size and dense clustering, this study introduces the MBCD, which includes nine species of bacteria, 310 images, and 23,524 colonies Based on this dataset, an improved detection network named Colony-YOLO is proposed. Firstly, StarNet is applied to the backbone network of Colony-YOLO, enhancing feature extraction while minimizing computational demands, which makes Colony-YOLO more suitable for deployment in mobile applications, aligning it with real-world usage scenarios. Secondly, the C2f-MLCA module is designed and implemented within the neck network to further boost the model’s feature extraction capabilities and performance in colony detection. Lastly, the Shape-IoU loss function is leveraged for its focus on the shape and size of bounding boxes in loss calculation, resulting in improved accuracy in bounding box regression. Additionally, an MBCD, which includes nine species of bacteria, 310 images, and 23,524 colonies, is introduced to train the model for the detection of the mulberry bacterial blight colonies.

Comparative experiments on the ADBCs and MBCDs demonstrate that Colony-YOLO significantly outperforms mainstream models such as Faster-R-CNN, YOLOv5n, YOLOv8n and YOLOv10n, achieving mAPs of 91.1% and 96.1%, which are 4.4% and 4.8% higher than the baseline. Meanwhile, Colony-YOLO achieved lightweight FLOPs and Params values of 6.5 G and 2.21 M in MBCD, respectively, which are 1.8 G and 0.8 M lower than the baseline. Additionally, the size of the weight file has been reduced to 4.7 MB. Smaller weight files are more beneficial for saving storage space, reducing model loading time, and facilitating deployment on resource-constrained devices. Visualization results further validate the model’s robustness in dense colony regions and low-contrast scenarios, enabling accurate detection of challenging tasks such as micro-colonies and adhered colonies. This provides reliable technical support for the early diagnosis and precise prevention of mulberry blight. Finally, a cross-platform intelligent detection system based on the Colony-YOLO model is designed and developed, enabling real-time image acquisition and processing on mobile devices to automatically detect and count colonies, thus providing a convenient and efficient solution for rapid on-site detection.

Future work will focus on expanding multi-source datasets to enhance the model’s generalization capability and exploring deployment optimization on mobile terminal devices, further promoting the practical application of intelligent monitoring for agricultural diseases.

## Figures and Tables

**Table 1:** Summary of bacterial species and infections.

Bacterial Species (Abbr.)	Reported Infection
*Pseudomonas syringae* (SY)	Mulberry bacterial blight [39]
*Pantoea ananatis* (PA)	Mulberry bacterial wilt [40]
*Pectobacterium parvum* (PP)	Potato bacterial soft rot [41]
*Klebsiella grimontii* (KG)	Hemorrhagic diarrhea [42]
*Pectobacterium carotovorum* (PC)	Cruciferous plants tuber soft rot [43]
*Stenotrophomonas maltophilia* (SM)	Zea mays L. seedling soft rot disease [44]
*Xanthomonas campestris* (XAN)	Cruciferous vegetables black rot [45]
*Pseudomonas fuwa* (FU)	Zanthoxylum spp. black rot
*Bacillus* sp. (BL)	Mulberry rhizosphere bacteria [46]

**Table 2:** Hyperparameter settings.

Hyperparameters	Value
Training epoch	400
Batch size	8
Learning rate	0.001
IoU	0.7
Optimizer	SGD
Image size	640 × 640
Weight_decay	0.005
Momentum	0.937
Warmup_momentum	0.8
Workspace	4

**Table 3:** Results of ablation experiments.

Models	StarNet	C2f-MLCA	Shape-IoU	P/%	R/%	mAP/%	FLOPs/G	Params/M	Weights/MB
M0	-	-	-	89.4	89.8	91.3	8.1	3.01	6.3
M1	✓	-	-	89.9	90.7	91.4	6.5	2.22	4.7
M2	-	✓	-	91.6	91.3	92.7	8.1	3.01	6.3
M3	-	-	✓	90.5	91.1	91.6	8.1	3.01	6.3
M4	✓	✓	-	93.4	91.5	94.5	6.5	2.21	4.7
M5	✓	-	✓	92.8	91.2	93.8	6.5	2.22	4.7
M6	-	✓	✓	94.3	92.4	95.4	8.1	3.01	6.3
M7	✓	✓	✓	**95.6**	**93.7**	**96.1**	**6.5**	**2.21**	**4.7**

Note: Where ✓ represents the use of this module, - indicates that this module is not used. Model M0 is the original YOLOv8n. Model M1 involves replacing the backbone network of YOLOv8s with StarNet. M2 incorporates the C2f-MLCA module. M3 replaces the loss function with Shape-IoU. M4, M5, and M6 represent StarNet + C2f-MLCA, StarNet + Shape-IoU, and C2f-MLCA + Shape-IoU, respectively. M7 represents StarNet + C2f-MLCA + Shape-IoU. The best results are presented in bold.

**Table 4:** Quantitative evaluation on different backbone network in MBCD.

Backbone	mAP/%	FLOPs/G	Params/M	Weights/MB
Original (yolov8)	91.3	8.1	3.01	6.3
MobileNet	88.4	22.5	8.72	16.7
ShuffleNet	84.6	16.4	6.38	12.9
FasterNet	86.7	10.7	4.17	8.6
StarNet	**91.4**	**6.5**	**2.22**	**4.7**

Note: The best results are presented in bold.

**Table 5:** Quantitative evaluation on loss function in MBCD.

IoU	P/%	R/%	mAP/%
CIoU	89.4	89.8	91.3
SIoU	87.2	86.4	90.9
GIoU	85.6	85.4	86.7
EIoU	90.0	87.5	90.1
Shape-IoU	**90.5**	**91.1**	**91.6**

Note: The best results are presented in bold.

**Table 6:** Quantitative evaluation of different model.

Datasets	Models	P/%	R/%	mAP/%	FLOPs/G	Params/M
Public dataset (ADBC)	Faster R-CNN	75.2	74.0	76.5	170.2	59.13
	YOLOv5n	81.2	79.9	82.6	**5.4**	2.56
	YOLOv8n	85.1	83.4	86.7	8.2	3.03
	YOLOv10n	86.3	82.1	87.4	7.1	2.75
	Colony-YOLO (Ours)	**90.3**	**88.5**	**91.1**	6.5	**2.21**
Private dataset (MBCD)	Faster R-CNN	76.1	73.8	78.5	165.9	58.72
	YOLOv5n	84.9	80.7	84.1	**5.4**	2.55
	YOLOv8n	89.4	89.8	91.3	8.3	3.01
	YOLOv10n	88.2	85.4	91.2	7.1	2.75
	Colony-YOLO (Ours)	**95.6**	**93.7**	**96.1**	6.5	**2.21**

Note: The best results are presented in bold.

## Data Availability

The public dataset used in this study are available in figshare at [https://doi.org/10.1038/s41597-023-02404-8], reference number [46]. These data were derived from the following resources available in the public domain: [https://figshare.com/articles/dataset/Annotated_dataset_for_deep-learning-based_bacterial_colony_detection/22022540/3]. (accessed on 15 October 2024). The private dataset presented in this study are available on request from the corresponding author.
